# Using patient-reported measures to drive change in healthcare: the experience of the digital, continuous and systematic PREMs observatory in Italy

**DOI:** 10.1186/s12913-020-05099-4

**Published:** 2020-04-16

**Authors:** Sabina De Rosis, Domenico Cerasuolo, Sabina Nuti

**Affiliations:** Management and He﻿althcare Laboratory﻿ (MeS), Institute of Management and EMbeDS, Scuola Superiore Sant’Anna, piazza Martiri della Libertà 33, Pisa, Italy

## Abstract

**Background:**

The use of Patient Reported Experience Measures (PREMs) has great potential in healthcare service improvement, but a limited use. This paper presents an empirical case of PREMs innovation in Italy, to foster patient data use up to the ward level, by keeping strengths and addressing weaknesses of previous PREMs survey experiences. The paper reports key lessons learned in this ongoing experience of action research, directly involving practitioners.

**Methods:**

The aim of this paper is to present the results of an ongoing action research, encompassing the innovation of PREMs collection, reporting and use, currently adopted by 21 hospitals of two Italian regions. The continuous and systematic PREMs collection has been implemented between 2017 and 2019 and includes: a continuous web-based administration, using web-services; an augmented and positive questionnaire matching standard closed-ended questions with narrative sections; the inclusion and benchmarking of patient data within a shared performance evaluation system; public disclosure of aggregated anonymized data; a multi-level and real-time web-platform for reporting PREMs to professionals. The action research was carried out with practitioners in a real-life and complex context. The authors used multiple data sources and methods: observations, feedback of practitioners, collected during several workshops and meetings, and analysis of preliminary data on the survey implementation.

**Results:**

A continuous and systematic PREMs observatory was developed and adopted in two Italian regions. PREMs participation and response rates tend to increase over time, reaching stable percentages after the first months. Narrative feedback provide a ‘positive narration’ of episodes and behaviours that made the difference to patients and can inform quality improvement actions. Real-time reporting of quantitative and qualitative data is enabling a gratifying process of service improvement and people management at all the hospitals’ levels.

**Conclusions:**

The PREMs presented in this paper has been recognized by healthcare professionals and managers as a strategic and positive tool for improving an actual use of PREMs at system and ward levels, by measuring and highlighting positive deviances, such as compassionate behaviours.

## Background

As the most important stakeholders in the healthcare system, patients can play a crucial role in healthcare services’ organizational design, management, and policymaking [1]. Monitoring and evaluating healthcare services in partnership with patients can provide key information to understand what works, inform quality improvement actions, and increase the value produced to patients [2]. Public disclosure and proper dissemination of patient data within healthcare organizations' performance evaluation systems can foster patient-driven changes in healthcare [3, 4]. This can positively impact organizations’ performance [5], and health professionals’ culture and behaviours [4, 6], on the basis of various mechanisms, such as the reputational lever [7].

The patient perspective measured in terms of experience provides clear factual and reliable results that can stimulate and inform quality improvement actions [2, 8]. Patient Reported Experience Measures (PREMs) can effectively measure quality of care and patient-centeredness [2, 9–15]. The use of PREMs data can determine positive modifications to administrative practices in healthcare and to the most important domains of patient experience [6, 16] to re-organize and transform healthcare [10, 17]. In addition, patient surveys can be used to introduce and evaluate new services and innovations [18–20].

The use of PREMs as institutional accreditation [21] and performance indicators within multi-dimensional performance evaluation systems can be powerful levers in orientating the efforts of healthcare organizations and professionals towards a more patient-centered and integrated care [3].

Despite this evidence, the use of PREMs data is still to be improved, and their full potential has yet to be exploited [10, 22–25]. A key question remains in how to reduce the distance between potential usefulness and the actual successful use of PREMs in healthcare. This paper presents the results of an ongoing action research, and suggests methods for improving the use of patient-data by solving several of the most important issues related to the collection and reporting of PREMs data. The innovations in the PREMs methodology presented in this paper are aimed at improving the use of PREMs data both at the system-level, by their integration into a multi-dimensional performance evaluation system, and up to the individual ward, by supporting the adoption of PREMs in the day-by-day operational management by healthcare professionals.

### Issues and barriers for PREMs use

Recently, Gleeson and colleagues [16] and Flott and colleagues [26] have drawn evidence on the use of patient-reported measures. Several issues and barriers in using patient-data for quality improvement actions are reported in the following paragraphs.

#### Sampling and data collection

Sampling is a cost-effective and easy method to carry out population surveys. Conversely, collecting patient experience data from a broader patient population [9, 26], on a regular basis [27] or continuously [10], can improve the credibility and value of collected data. However, a systematic and continuous approach can be expensive and difficult to achieve [27].

The various methods for administering surveys present different strengths and weaknesses [23, 26]: postal surveys are very time-consuming and expensive, and imply a high risks of data entry errors; in addition to the aforementioned limitations, telephone surveys require brief questionnaires, and can produce interviewer-bias; web-surveys also need brief questionnaires, and must consider the use of different digital devices in accessing the online questionnaire [23]. Recently, it was found that, in comparison to other survey administration methodologies, a web-based methodology does not increase the level of selection bias [28, 29], due to a general increase in smartphones and Internet use by older people. In addition, web-based surveys do not produce significant differences in patient reported data [28, 30].

#### Timeliness of data reporting

An advanrage of web-based surveys is the reduction of the time needed to collect and report patient data. Timeliness is, conversely, a barrier in using patient data obtained by postal and telephone surveys [23, 31]. Recently, some healthcare organizations have introduced web-based PREMs including real-time feedback systems [32–34], to give a quick response to patients’ issues and to foster the culture of patient-driven quality improvement [31, 33]. Currently, this methodology has been mainly used to capture patients’ on-site feedback, and rarely to collect post-treatment or post-episode feedback in a regular and continuous manner [23, 32–34]. At present, patients can also voluntarily give feedback on their experiences using social networks [35], and on institutional websites, if any (i.e. NHS Choices- www.nhs.uk) [36]. However, this source of information presents several criticisms, such as the impossibility to know the representativeness of the comments, and an inability to use these data for benchmarking or monitoring trends [23].

#### Type of data

Providing measurable and actionable indicators from patient surveys is fundamental to the success of survey [26]. Clinicians are more likely to use patient reported measures if they know these are important to the patients for whom they are responsible for [23, 26]; this has several important implications on the typology of data collected.

Patient surveys usually include standard measures of patient experience to allow the benchmarking within and among healthcare organizations [3, 37, 38]. However, standard closed-ended questions can fail to catch detailed information on patient experience [39]. In addition to standard structured surveys, narrative information provides a ‘supplemental value’, therefore enriching the quantitative results by detail and context [10, 40, 41]. By adding anecdotal sections to standard questionnaires, patient surveys can improve while remaining suitable for the internal and external benchmarking. Secondly, when patients data are reported in an aggregated format at the provider level, they fail to support improvements relevant to the individual service or ward [26].

#### Level of data aggregation, benchmarking and timeliness

Healthcare professionals prefer micro-level data reported by patients cared in their ward, in order to link this data to their ‘real-life’ work [9, 26, 39]. Specific data at the ward level can help identify problems that can immediately be addressed by practical improvements [23]. Furthermore, this specific level of data reporting can indicate to managers the variability of performance between wards and services [42]. This internal benchmarking is an important activity for improving the patient experience [23] and reducing internal variation [43–46]. The comparison of data among homogeneous wards is also very relevant. Medical and surgical care need separate analyses and comparisons, due to a difference in factors affecting the patient satisfaction [47].

As mentioned above, timeliness is another factor affecting the use of patient data for quality improvement [48]. Having patient feedback several months after the patient experience occurred can decrease health professionals’ interest in the patient data and, thus, their likelihood to take action. Healthcare workers prefer close-in-time or real-time feedback, because they make it easier to remember the circumstances of complaints and to address them in a timely manner [39, 49, 50]. Regular patient experience measurement, monitoring, and reporting are relevant in achieving and sustaining more substantial changes in healthcare [23, 51].

#### Data reporting

Finally, the method of communicating data to managers, clinicians and staff is crucial: the volume and variety of patient data collected and reported can be overwhelming and lead to fatigue, adversely affecting their perception of the usefulness and the actual use of PREMs [39]. Clear PREMs data reporting, communication and sharing arrangements are needed [23]. Another key challenge in the use of patient data is the lack of data integration. When patient data are isolated from other relevant data, it is difficult to understand the relationship between effectiveness, safety and experience at the patient level [26]. The integrated and synchronous reporting of patient data with indicators from other sources increases data value to professionals and managers [3], as in the multi-dimensional performance evaluation systems [3].

The above issues can contribute to explaining the limited use of PREMs data to improve healthcare service delivery, and to monitor the implementation and effectiveness of improvement efforts [16].

## Methods

### Study design

The aim of this paper is to present the results of an ongoing action research [52], which is appropriate when a new approach is built or implanted on an existing system [53], such as the traditional way of PREMs’ collection, reporting and use. The action research was carried out by practitioners and researchers in order to solve the problem of patient-data underuse and to improve adoption of data for informing strategies and actions both at the system and at the individual ward levels. In this paper, the authors describe the innovations adopted in the PREMs methodology in a real-life and complex context, where participants, both researchers and practitioners, have taken part in the investigation, by reviewing, evaluating and improving the practice.

The authors used an approach based on multiple sources and methods. Observations of real-life events were combined with feedback by practitioners, collected during workshops and meetings.

Descriptive statistics were performed on the preliminary data by using the STATA.15 software.

### Study setting

Italy has a public and universal healthcare system, financed by general taxation, largely free of charge at the point of delivery, and regionally managed. Healthcare services are provided by Local Health Authorities (LHAs), which directly manage hospitals among other suppliers, and Teaching Hospitals (THs), which have also the mission of training healthcare professionals and advancing research. The context of this paper is the Italian regions of Tuscany and Veneto. These regions share a common healthcare Inter-Regional Performance Evaluation System (IRPES), designed by the Management and He﻿althcare Laboratory﻿ (MeS) of the Scuola Superiore Sant’Anna and implemented for the first time in 2005, in Tuscany. The IRPES indicators measure and monitor quality, efficiency, appropriateness, as well as patient and staff satisfaction [3, 43, 54, 55]. Currently, twelve Italian regions have adopted the IRPES on a voluntary basis. The IRPES includes multi-dimensional indicators from administrative data as well as from surveys to healthcare organizations’ personnel and to the patients. The integration of indicators reported by patients has been an example of patient data used to measure the quality of care [3], and to improve the doctor-patient communication, meant as a dimension of the patient experience [4]. However, patient experience surveys have been usually administered every two years. The last internal organizational climate survey in Tuscany and Veneto showed that, despite the availability of patient data, a significant proportion of healthcare professionals are not familiar with this data, and that they are unsatisfied with the acknowledgment and the value given to their competences, skills and work.

Between 2017 and 2019, Tuscany and Veneto implemented a continuous and systematic digital collection and real-time reporting of PREMs regarding inpatient care experience, called PREMs Observatory and carryed out in collaboration with the MeS. This PREMs model was first implemented in 2017 by eight Tuscan public accredited private hospitals, which are part of the Italian Association of Private Hospitals (AIOP). In 2018, 18 public hospitals in Tuscany and seven in Veneto joined the PREMs program. Between January and February 2019, another two LHA hospitals and one teaching hospital joined the Observatory. At the time of this research (March 2019), the program had completed the design phase and had begun the data collection from a total of 26 healthcare suppliers that refer to 21 hospitals. In tables and figures included herein, the hospitals directly managed by LHAs are indicated by the code H, while the Teaching Hospitals by the code TH. Overall, six healthcare organisations were involved, including both LHAs and THs. The full deployment of the PREMs Observatory in Tuscany and Veneto is expected in 2019. Another Italian region is also working with the MeS to implement the PREMs model in 2020.

#### Practitioners’ involvement

The action research is based upon the identification of a need for improvement in PREMs use by the same practitioners. As mentioned above, managers and healthcare professionals reported a low knowledge and use of PREMs in answering to the last organizational climate survey. The innovations to the PREMs methodology have been proposed by the researchers, then defined in detail and implemented with policy makers and technicians in Tuscany and Veneto, and top-level and mid-level health care managers, including nurses coordinators and ward managers. Their involvement in the pilot stage of this innovative PREMs was continuous, leading to a ‘feedback loop’ [56]. In the early stages, their involvement encompassed the monitoring of organizations’ implementation choices, the health care workers’ response to the innovations (also measured in terms of participation rate and early impacts on their behaviors, procedures and practice), and the patients’ response. After the implementation of the PREMs Observatory, the practitioners’ involvement has also been aimed at investigating new opportunities of PREMs data use in the day-by-day practice.

In the Table 1, the number of meetings and participants were reported by health care supplier, healthcare organization and region. Researchers organized and participated in two typologies of meetings with practitioners: meetings with managers at different levels, mainly at the regional and healthcare organization levels; workshops, focus groups and collective events with an extended participation of mainly middle managers, wards managers, and nurse coordinators. The researchers involved into the meetings at least one representative of the managerial group and one of the nurses coordination group, for each TH or hospital directly managed by a LHS, and for each ward involved into the PREMs. The workshops were mainly aimed at sharing the preliminary results of the PREMs Observatory, in terms of implementation and, if available, also in terms of patients’ feedback. The meetings with managers were also held during the design phase, in addition to the implementation phase, with the aim of sharing decisions on the design of the initiative and monitoring its development. Participants to these groups addressed the following topics: how to increase the practitioners’ involvement within each ward; how to improve the communication of PREMs to patients and increase their willingness to participate; how to increase the use of PREMs to inform quality improvement actions; how to integrate healthcare practitioners’ evaluations with those from the concrete experiences of patients; how to provide high quality services as perceived by patients. During these events, field notes were independently collected by a group of eight trained and/or experienced researchers. Almost three researchers took part to each event. Two members of the research group also participated in the early phase of the PREMs Observatory design. The researchers focused on the same aspects, related to the issues and barriers described above: these aspects were systematically and carefully observed. After each event, the observers participated in debriefing sessions for comparing and discussing their notes, and verifying their consistency.

**Table 1:** Number of encounters (meeting with managers and workshops with top. Middle and wards managers. Healthcare professionals and nurse coordinators) by Region. healthcare organisation and hospital. Note. Time-period: March 2018–February 2019

***Region***	***Healthcare Organisation***	***Directly Managed Hospital***	***Meetings with managers (n)***	***Participant managers (total number - mean - SD - min - max)***	***Workshops and collective events (n.)***	***Participants to workshops and collective events (total number of participants - mean - SD - min - max)***
***Region A***			3	*No. 15- Mean 5.00- Std.Dev. 0.00- Min. 5- Max. 5*		
	**LHA 1**		3	*No. 10- Mean 3.33- Std.Dev. 1.53- Min. 2- Max. 5*		
	**LHA4**		1	*16*		
	**LHA5**		1	*15*		
***Region B***			2	*No. 14- Mean 7.00- Std.Dev. 5.66- Min. 3- Max. 11*	*1*	123
	**LHA2**		3	*No. 27- Mean 9.00- Std.Dev. 10.39- Min. 3- Max. 21*	*1*	16
		**H2.3**			*1*	25
	**LHA3**		3	*No. 27- Mean 9.00- Std.Dev. 6.93- Min. 5- Max. 17*	*1*	19
	**TH1**				*1*	9
	**TH2**		4	*No. 16- Mean 4.00- Std.Dev. 1.15- Min. 3- Max. 5*	*3*	No. 103- Mean 34.33- Std.Dev. 6.35- Min. 27- Max. 38
	**TH3**		1	*5*		
	**TH4**		1	*6*		
	**TH5**		1	*3*		
	**LHA6**		1	*5*		
***OVERALL***			***24***	***No. 159- Mean 6.63- Std.Dev. 5.24- Min. 2- Max. 21***	***8***	***No. 295- Mean 36.88- Std.Dev. 36.24- Min. 9- Max. 123***

#### Description of the intervention

The innovations illustrated in this paper combine the strengths of traditional survey methods with advancements in the PREMs methodology aimed at overcoming some limitations that the same methods pose.

Key aspects retained from the previous PREMs experiences are: the standard questionnaire of patient experience evaluation; the inclusion of PREMs in the multi-dimensional health care performance evaluation system (IRPES); benchmarking within and among healthcare organizations/hospitals using the quantitative patient data from the standard questions; public disclosure of the PREMs results.

The main innovations implemented in the PREMs to overcome some of the limitations of the traditional approach are the followings:

- Reducing sampling limits by systematically proposing participation in PREMs survey to all hospitalized patients: a sort of census survey, which allows for a detailed data reporting, particularly in reference to wards;
- Adopting an augmented traditional survey that combines standard closed questions with narrative sections to capture what was relevant in the individual patient’s experience;
- Using a web-based questionnaire and a fully digital administration method, in order to limit the costs of the survey and to avoid risks of errors in the data collection and data entry;
- Collecting data regarding the hospitalization directly from patients immediately following their discharge;
- Providing a real-time online tool of PREMs reporting that completely avoids a time gap between data collection and reporting, allows for benchmarking among hospitals, departments and specialties, and allows different access levels to level-specific data, from the ward to the regional level.

#### Patient participation process

The PREMs were implemented as a systematic, continuous and census-like survey. In Tuscany and in Veneto, during the hospital stay, every patient is informed by the healthcare workers about the possibility to take part in the survey. Informational material, such as posters and flyers, are kept available in the participating wards. An informative document explaining the initiative, its goals and the data protection and privacy policy is given to each patient by healthcare personnel. It includes information on the anonymity of the survey, because no identification data are collected, and answers to closed-ended questions are reported in aggregated form. The informative document explains that responses to the open-ended questions are voluntary and individually reported to healthcare managers and professionals, without making change or anonymizing them. The informative document asks patients not to add identifying information, because names or other details that can allow identifying patients or healthcare professionals are not deleted. The same information is mentioned in the questionnaire, at the beginning of every open-ended questions’ section. The informative document also clearly explains that data are reported by the researchers to the healthcare organizations for internal use (i.e., quality improvement actions), according to the different levels of responsibility, and that data will be used for the annual performance evaluation within IRPES.

Patients can decide to take part to the survey, and leave their contact information (email address and/or mobile phone number, or the caregiver’s contact information) for sole purpose of the PREMs survey. 24 h after the discharge, the patient receives via text message and/or email a unique personal link to access the online questionnaire. A reminder email/text message is sent to the patient five days after the invitation. The link expires in one month after it has been sent.

#### Data collection

The administration method is based on web services. PREMs’ continuity is based on the modification of the electronic medical record (EMR) software, that requires the inclusion in the EMR of a specific PREMs section, where healthcare workers input patient contact information for the purposes of the survey. The list of patients’ telephone numbers and email addresses is collected through a continuous data flow based on an Application Programming Interface (API) established between each hospital’s EMR and the MeS server. This data flow also contains some pre-existing EMR data, namely: the hospital provider; date and ward of admission; date and ward of discharge; wards of transition during the hospitalization; additional patient data, if available (citizenship, level of education, residence).

The data flow does not contain the first or last name of patients. If the patient does not wish to participate, contact information is not collected in the PREMs section of the EMR, and, as a consequence, the record is automatically not included into the data flow.

The delivery via e-mail and/or text message of the invitation to take part in the survey is automatic and based on web services enabled by the data flow from the hospitals. The questionnaire is only available online. Patient’s contact information is automatically deleted five days after the reminder, or right after the completion of the questionnaire, whichever occurs first.

As previously mentioned, healthcare workers and professionals are in charge of informing patients of the survey and collecting their contact details. In Tuscany, two indicators measuring participation and response rates were added in the IRPES. Targets and incentives are linked to these two indicators, with the aim to avoid biases attributable to practitioners by discouraging opportunistic behaviours (i.e. selection of patients), preventing scepticism or embarrass of in proposing the participation to PREMs and encouraging to enroll all patients [57].

#### Questionnaire

Four aspects of the PREMs questionnaire were revised with regard to the original version used in Tuscany in previous PREMs experiences [4, 21]:

i) the general approach of the questionnaire;
ii) the typology of questions;
iii) the order of the questions;
iv) the length of the questionnaire.

Each element is addressed below.

(i) The closed-ended questions are aimed at investigating the standard dimensions of the patient experience (i.e. responsiveness, comfort) [58]. Because people naturally tend to pay more attention to and are more influenced by the negative aspects of their experience, the questions were reviewed in order to move the focus from the negative to the positive aspects of the experience and mitigate the so-called ‘negativity bias’ [59, 60]. For instance, the questions on comfort ask if the ward was quiet, not noisy. In the same way, the Likert-scales were ordered from the most positive to the most negative option. The questionnaire is not aimed at simply highlighting what does not work; it mainly draws attention to what works, on best practices and the practical solutions that can help healthcare workers improve patient experience [61, 62].
(ii) The adopted questionnaire combines the standard questions with a narrative section to describe what and who were relevant to the patient during the hospital stay. This augmented survey allows patients to an in-depth and personal assessment and description of their experience. Patients can indicate the healthcare workers’ behaviours that met their needs, for example in terms of compassion, respect and humanity: these aspects are not well addressed by the closed-ended questions [63]. The measures are self-reported by patients using the online questionnaire after discharge: this avoids the risk of biases related to the interviewer and of completing the survey during the care experience. The online questionnaire may be accessed by any type of device having an Internet connection (i.e. computer, tablet, or smartphone): its design is completely responsive.
(iii) The questions, traditionally grouped in homogeneous domains (i.e. communication with personnel; pain management), have been re-organized in order to follow the various phases of thepatient experience journey during the hospitalization. The questionnaire was divided into sections, accordingly: the first section refers to the admission process; the second, to the experience in the ward; the third, to the discharge phase. The first question refers to the individual actually filling in the questionnaire, in order to understand if the patient was assisted by a caregiver. The health-related and socio-demographic characteristics of the respondents are collected in the last section (see Additional file 1).
(iv) A briefer questionnaire was used with respect to the questionnaires used in previous PREMs experiences [21], in order to reduce the burden of the questionnaire completion, and considering that the administration is web-based. The survey is comprised of thirty-three closed-ended questions and six open-ended questions. The closed-ended questions are mandatory and include questions regarding the socio-demographic characteristics of the patient and of the caregiver, if someone is assisting the patient in responding. Thirteen of the closed-ended questions are Likert-scale questions; the remaining are single and multiple choice questions. Responding to the open-ended questions is not mandatory on the behalf of the patient.

#### The double reporting of data

**Figure 1:**
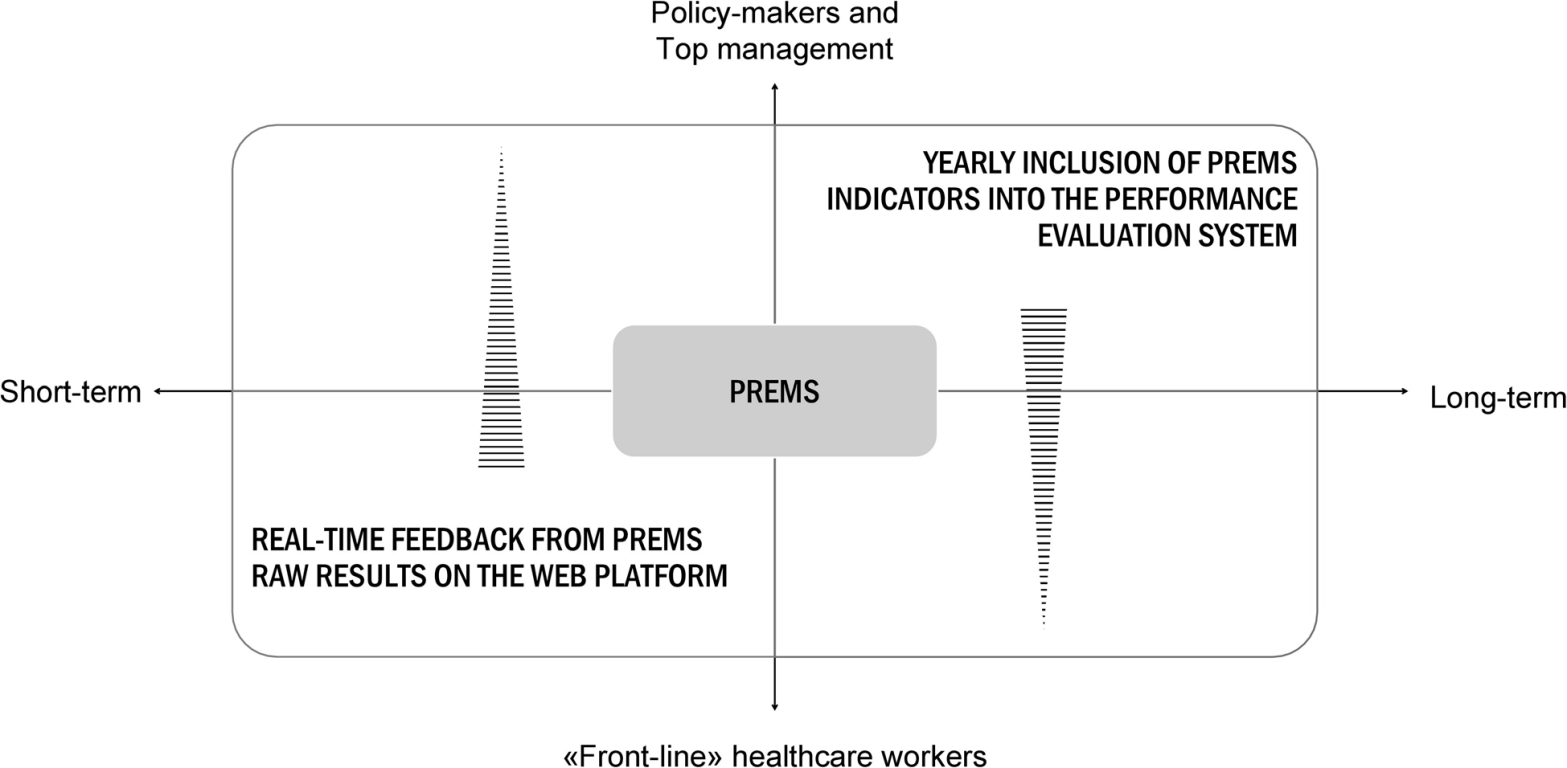
**Fig. 1** A conceptual framework of PREMs data use at the different levels of the healthcare system and organization

The patient survey is completely managed by the MeS, which, as an external third-party, assures the confidentiality of the survey, and provides a yearly benchmarking of results within the IRPES (Fig. 1). Data collected through PREMs surveys are used to calculate performance indicators one time a year, as for previous surveys [3]. Indeed, the quality of care from the patients’ perspective was operationalised over time, in previous survey initiatives held in Tuscany region. These indicators are integrated in the yearly IRPES, together with data from other sources (i.e. administrative data). PREMs indicators are generally calculated at the hospital level, mainly for the use of policy-makers and top managers (Fig. 1). The annual indicators are calculated using the data collected within the PREMs Observatory the previous year. Because the most of healthcare organizations joined the initiative on 2019, the first indicators from the PREMs Observatory will be available in 2020. Generally, multi-level models are performed to calculate indicators, which requires a sufficient amount of data from each provider. Because of the census-like approach of the survey, the researchers expect to reach an adequate size of respondents. However, while the size of the respondents’ population was not perceived as an issue in the day-by-day managerial use of the real-time reported data, managers and professionals, whose performance is evaluated on the basis of the IRPES indicators, stated that the representativeness of PREMs data is a relevant and potentially critical aspect. To this end, size and characteristics of the surveyed population will be compared to those of hospitalised patients in the administrative data flow, to verify whether or not those who participated in the survey are representative of the reference population. If the analysis will produce a negligible deviation, the researchers will perform a further sensitivity analysis [64]. Otherwise, in the case of a significant selection bias, the researchers will proceed weighting data at the hospital level [65–68]. In any case, in order to correctly perform the multi-level analysis, providers with less than 30 respondents will be excluded from the analysis. A multi-level case-mix adjustment is performed on individual data, to ensure comparisons among hospitals with patient populations having different compositions and characteristics, considering the hierarchical organization of data [67–69]. Generally, the evaluation is also performed by grouping some typologies of hospitals, in order to compare more similar organizations (i.e., teaching hospitals, paediatric hospitals) [47, 67]. Public disclosure is one of the key characteristics of the IRPES, not only for transparency reasons, but also to improve performance [7, 70].

Among the innovations encompassed by the PREMs Observatory, the day-by-day online reporting of real-time data is one of the most important. This data reporting is in addition to the annual evaluation that is in benchmarking as mentioned above, but it vertically permeates the healthcare organisations form the ward to the system (Fig. 1). Indeed, PREMs results are available in a raw form directly to practitioners. By developing specific web services, the patient measures and comments reported on the questionnaire are instantly available on an online platform. Data are reported aggregated and anonymised, without risks of errors in the data entry process and without additional costs over time. In the beta version of the web platform, patients’ answers were reported in graphics and tables. The formats were chosen on the basis of the findings emerged from previous experimental studies on readability and clarity of the performance data representation [71]. In the graphic representation of questions with five ordinal levels, five evaluation tiers were associated with different colours of the performance evaluation adopted in the IRPES, from dark green (excellent performance) to red (poor) [43], which looked familiar to practitioners. In the tables, the number and percentage of respondents for each response option are reported, as well as the total number of respondents to each question. On the web platform, data are not weighted, nor risk adjusted, because of they are real-time updated. They are reported raw, as collected. The characteristics of respondents may be also viewed on the web platform, formatted in tables and graphs. Results can be filtered by healthcare organization, hospital, specialty and ward, based on the access permissions. Results can be also consulted for time-periods, on the basis of the selection of the user on the web platform. A threshold of 20 returned questionnaires was established, in order to consult results on the web platform, even though the Italian privacy guidelines report a minimum threshold of three observations (Italian Law 515/2018, Art. No. 5) [72]. The threshold is applied also when selecting time-periods: if less than 20 answers are available for the period, an informative massage is displayed instead of data. Currently, data are presented on the web platform on a cumulative basis. Using the functionality aimed at selecting specific time-periods, old data can be accessed with new data, for instance to compare patients’ feedback over time. The answers to the open-ended questions are reported individually, and are not anonymized. They can be navigated by key words through an internal search engine, for going deeper in the patient stories. Because the online front-end is based on API technologies, it is possible to integrate PREMs data with other digital business intelligence tools used by the healthcare organizations.

Real-time reported data are not made available to the public. Conversely, PREMs indicators encompassed by the annual evaluation are publicly disclosed on the IRPES website.

## Results

### Participation and response rates

The participation rate, measured as the ratio between the number of invited patients and the number of discharged patients, has increased over time (Table 2).

**Table 2:** Patients to accepted to take part to PREMs over time: absolute numbers of participants and participation rate, as ratio between patients who accepted to take part to PREMs and discharged patients, and relative mean, minimum, maximum and standard deviation)

			Participants by month				Participation rate by month			
Total by month	Total Participants	Mean Participation Rate	Mean	Min	Max	SD	Mean	Min	Max	SD
**mar-18**	3	1%	3	3	3	–	1%	1%	1%	–
**apr-18**	88	43%	88	88	88	–	43%	43%	43%	–
**may-18**	146	71%	146	146	146	–	71%	71%	71%	–
**jun-18**	147	71%	147	147	147	–	71%	71%	71%	–
**jul-18**	172	35%	86	31	141	77.78	35%	1%	68%	48%
**aug-18**	280	32%	140	124	156	22.63	32%	3%	60%	40%
**sep-18**	359	31%	179.5	11	242	88.39	31%	5%	57%	37%
**oct-18**	746	24%	149.2	67	241	72.30	23%	5%	66%	29%
**nov-18**	1919	20%	191.9	1	583	157.60	23%	4%	61%	20%
**dec-18**	2996	14%	166.44	11	772	189.97	19%	1%	75%	21%
**jan-19**	4207	23%	210.35	29	726	179.58	26%	1%	72%	19%
**feb-19**	4708	26%	224.19	6	810	200.82	27%	1%	81%	1%
**Total**	15,771	–								
**Mean**	1173.17	33%								
**Min**	3	1%								
**Max**	4200	71%								
**SD**	1510.83	21%								

Note. Time-period: March 2018–February 2019

Overall, the trend of the average participation rate for LHAs depends mostly on the PREMs deployment date in their directly managed hospitals. The first start-up period was characterised by a variable percentages of patients’ participation, mainly due to technical fine-tuning activities. Thereafter, the PREMs participation rate became stable around 30%, and in some cases overcoming 80% of the inpatient population (Table 3). In the first year of the PREMs Observatory, 15,771 patients provided their contact information specifically to be invited to the PREMs initiative.

**Table 3:** Participants (a) and Participation rate (b) by hospital over time

**a. Patients to accepted to take part to PREMs by hospital and over time**																	
**Hospitals**	**mar-18**	**apr-18**	**may-18**	**jun-18**	**jul-18**	**aug-18**	**sep-18**	**oct-18**	**nov-18**	**dec-18**	**jan-19**	**feb-19**	**Total by hospital**	**Mean by hospital**	**Min by hospital**	**Max by hospital**	**SD by hospital**
**H1.1**									77	63	52	56	248	62.00	52.00	77.00	10.98
**H1.2**									152	172	150	145	619	154.75	145.00	172.00	11.87
**H1.3**								204	583	772	726	810	3095	619.00	204.00	810.00	247.42
**H1.4**								99	230	226	181	187	923	184.60	99.00	230.00	52.73
**H1.5**								67	274	333	331	304	1309	261.80	67.00	333.00	111.51
**H2.1**									136	286	292	316	1030	257.50	136.00	316.00	82.03
**H2.2**									133	202	203	217	755	188.75	133.00	217.00	37.79
**H2.3**										27	144	165	336	112.00	27.00	165.00	74.36
**H2.4**											154	240	394	197.00	154.00	240.00	60.81
**H3.1**										68	116	83	267	89.00	68.00	116.00	24.56
**H3.2**										11		6	17	8.50	6.00	11.00	3.54
**H3.3**										12	54	114	180	60.00	12.00	114.00	51.26
**H3.4**									1	359	529	523	1412	353.00	1.00	529.00	247.53
**H3.5**										38	81	86	205	68.33	38.00	86.00	26.39
**H3.6**										38	247	170	455	151.67	38.00	247.00	105.70
**H3.7**											100	96	196	98.00	96.00	100.00	2.83
**H3.8**										45	59	85	189	63.00	45.00	85.00	20.30
**H3.9**										11	179	340	530	176.67	11.00	340.00	164.51
**TH1**											29	32	61	30.50	29.00	32.00	2.12
**TH2**					31	156	242	241	207	206	454	580	2117	264.63	31.00	580.00	173.01
**TH3**	3	88	146	147	141	124	117	135	126	127	126	153	1433	119.42	3.00	153.00	40.47
***Total***	***3***	***88***	***146***	***147***	***172***	***280***	***359***	***746***	***1919***	***2996***	***4207***	***4708***	***15,771***				
**b. Participation rate (ratio between patients who accepted to take part to PREMs and discharged patients) by hospital and over time**																	
**Hospitals**	**mar-18**	**apr-18**	**may-18**	**jun-18**	**jul-18**	**aug-18**	**sep-18**	**oct-18**	**nov-18**	**dec-18**	**jan-19**	**feb-19**	**Mean by hospital**	**Min by hospital**	**Max by hospital**	**SD by hospital**	
**H1.1**									4%	4%	3%	3%	4%	3%	4%	0.01	
**H1.2**									13%	16%	13%	12%	14%	12%	16%	0.02	
**H1.3**								15%	49%	75%	72%	81%	58%	15%	81%	0.27	
**H1.4**									24%	27%	19%	21%	23%	19%	27%	0.03	
**H1.5**								6%	26%	39%	36%	34%	28%	6%	39%	0.13	
**H2.1**									12%	23%	32%	37%	26%	12%	37%	0.11	
**H2.2**									12%	18%	25%	29%	21%	12%	29%	0.07	
**H2.3**										4%	22%	26%	17%	4%	26%	0.12	
**H2.4**											21%	27%	24%	21%	27%	0.05	
**H3.1**										5%	11%	8%	8%	5%	11%	0.03	
**H3.2**										18%		9%	13%	9%	18%	0.06	
**H3.3**										2%	9%	22%	11%	2%	22%	0.10	
**H3.4**										19%	34%	36%	30%	19%	36%	0.09	
**H3.5**										12%	29%	33%	25%	12%	33%	0.12	
**H3.6**										4%	32%	23%	20%	4%	32%	0.14	
**H3.7**											44%	43%	44%	43%	44%	0.01	
**H3.8**										9%	16%	21%	15%	9%	21%	0.06	
**H3.9**										1%	25%	47%	24%	1%	47%	0.23	
**TH1**											1%	1%	1%	1%	1%	0.00	
**TH2**					1%	3%	5%	5%	4%	4%	13%	17%	7%	1%	17%	0.06	
**TH3.1**	1%	43%	71%	71%	68%	60%	57%	66%	61%	62%	68%	45%	56%	1%	71%	0.20	
***Total***	***1%***	***43%***	***71%***	***71%***	***35%***	***32%***	***31%***	***23%***	***23%***	***19%***	***26%***	***27%***					

Note. Codes: *H* hospital directly managed by a LHA; *TH* teaching hospital. Time-period: March 2018–February 2019

The response rate to the web-based survey, measured as the ratio between the number of respondents and the number of patients contacted, also presented a slight increasing trend that became stabilized at around 30%, after the initial deployment phase (Table 4). While the participation rate has been visibility affected by the entering of new organisations in the initiative, the response rate appeared less variable.

**Table 4:** Patients to answered to the PREMs questionnaire over time: absolute numbers of respondents and response rate, as ratio between patients who responded and patients who accepted to take part to PREMs, and relative mean, minimum, maximum and standard deviation)

			Respondents by month				Response rate by month			
Total by month	Total Respondents	Mean Response Rate	Mean	Min	Max	SD	Mean	Min	Max	SD
**mar-18**	1	33%	1	1	1	–	33%	33%	33%	–
**apr-18**	31	35%	31	31	31	–	35%	35%	35%	–
**may-18**	58	40%	58	58	58	–	40%	40%	40%	–
**jun-18**	59	40%	59	59	59	–	40%	40%	40%	–
**jul-18**	63	36%	31,5	11	52	28,99	36%	35%	37%	1%
**aug-18**	92	34%	46	40	52	8,49	34%	26%	42%	12%
**sep-18**	119	35%	59,5	49	70	14,85	35%	29%	42%	9%
**oct-18**	237	35%	47,4	30	83	20,55	35%	17%	45%	11%
**nov-18**	797	44%	88,56	28	243	73,87	42%	21%	89%	23%
**dec-18**	779	29%	43,28	1	120	38,76	28%	9%	47%	11%
**jan-19**	1093	28%	13	9	156	42,50	27%	12%	39%	8%
**feb-19**	1287	30%	61,29	2	197	50,08	29%	12%	37%	12%
**Total**	4616	–								
**Mean**	384,67	35%								
**Min**	1	28%								
**Max**	1287	44%								
**SD**	467,88	5%								

Note. Time-period: March 2018–February 2019

From the implementation date through February 2019, more than 30 questionnaires were collected by almost each participating hospital. Only one hospital collected less than 30 questionnaires, which was chosen as the minimum number of observations for calculating the annual PREMs indicators. This was the case for a hospital in the initial phase of PREMs implementation (H3.2, n. 3 questionnaires). On average, 4616 questionnaires were collected by the hospitals in the first year (and for several hospitals, in the first few months) of the PREMs Observatory, with a mean of more than 380 questionnaires per month. As reported in the Table 5, there is a high variability among hospitals in the number of collected questionnaires, due to the hospital size and, as amentioned earlier, to the time of PREMs implementation.

**Table 5:** Respondents (a) and Response rate (b) by hospital over time

a. Patients who answered to the PREMs questionnaire by hospital and over time																	
**Hospitals**	**mar-18**	**apr-18**	**may-18**	**jun-18**	**jul-18**	**aug-18**	**sep-18**	**oct-18**	**nov-18**	**dec-18**	**jan-19**	**feb-19**	**Total by hospital**	**Mean by hospital**	**Min by hospital**	**Max by hospital**	**SD by hospital**
**H1.1**									31	23	13	19	86	21.5	13	31	7.55
**H1.2**									54	64	56	46	220	55	46	64	7.39
**H1.3**								83	128	87	84	95	477	95.4	83	128	18.82
**H1.4**								39	163	65	68	70	405	81	39	163	47.52
**H1.5**								30	243	118	130	99	620	124	30	243	76.96
**H2.1**									44	72	81	65	262	65.5	44	81	15.76
**H2.2**									28	61	43	54	186	46.5	28	61	14.39
**H2.3**										8	26	51	85	28.33	8	51	21.59
**H2.4**											39	87	126	63	39	87	33.94
**H3.1**										15	16	19	50	16.67	15	19	2.08
**H3.2**										1		2	3	1.5	1	2	0.71
**H3.3**										2	9	26	37	12.33	2	26	12.34
**H3.4**										120	116	173	409	136.33	116	173	31.82
**H3.5**										12	22	29	63	21	12	29	8.54
**H3.6**										18	87	54	159	53	18	87	34.51
**H3.7**											39	25	64	32	25	39	9.9
**H3.8**										17	17	25	59	19.67	17	25	4.62
**H3.9**										2	50	89	141	47	2	89	43.58
**TH1**											10	10	20	10	10	10	–
**TH2**					11	40	70	42	56	39	156	197	611	76.38	11	197	64.94
**TH3**	1	31	58	59	52	52	49	43	50	55	31	52	533	44.42	1	59	16.46
***Total***	***1***	***31***	***58***	***59***	***63***	***92***	***119***	***237***	***797***	***779***	***1093***	***1287***	***4616***				
**b. Response rate (ratio between patients who responded to PREMs and patients who accepted to particapate to PREMs) by hospital and over time**																	
**Hospitals**	**mar-18**	**apr-18**	**may-18**	**jun-18**	**jul-18**	**aug-18**	**sep-18**	**oct-18**	**nov-18**	**dec-18**	**jan-19**	**feb-19**	**Mean by hospital**	**Min by hospital**	**Max by hospital**	**SD by hospital**	
**H1.1**									40%	37%	25%	34%	34%	25%	40%	0.06	
**H1.2**									36%	37%	37%	32%	35%	32%	37%	0.03	
**H1.3**								41%	22%	11%	12%	12%	19%	11%	41%	0.13	
**H1.4**								39%	71%	29%	38%	37%	43%	29%	71%	0.16	
**H1.5**								45%	89%	35%	39%	33%	48%	33%	89%	0.23	
**H2.1**									32%	25%	28%	21%	26%	21%	32%	0.05	
**H2.2**									21%	30%	21%	25%	24%	21%	30%	0.04	
**H2.3**										30%	18%	31%	26%	18%	31%	0.07	
**H2.4**											25%	36%	31%	25%	36%	0.08	
**H3.1**										22%	14%	23%	20%	14%	23%	0.05	
**H3.2**										9%		33%	21%	9%	33%	0.17	
**H3.3**										17%	17%	23%	19%	17%	23%	0.04	
**H3.4**										33%	22%	33%	29%	22%	33%	0.07	
**H3.5**										32%	27%	34%	31%	27%	34%	0.03	
**H3.6**										47%	35%	32%	38%	32%	47%	0.08	
**H3.7**											39%	26%	33%	26%	39%	0.09	
**H3.8**										38%	29%	29%	32%	29%	38%	0.05	
**H3.9**										18%	28%	26%	24%	18%	28%	0.05	
**TH1**											34%	31%	33%	31%	34%	0.02	
**TH2**					35%	26%	29%	17%	27%	19%	34%	34%	28%	17%	35%	0.07	
**TH3**	33%	35%	40%	40%	37%	42%	42%	32%	40%	43%	25%	34%	37%	25%	43%	0.05	
***Total***	***33%***	***35%***	***40%***	***40%***	***36%***	***34%***	***35%***	***35%***	***42%***	***28%***	***27%***	***29%***					

Note. Codes: *H* hospital directly managed by a LHA; *TH* teaching hospital. Time-period: March 2018–February 2019

### PREMs as storytelling and quality improvement tool

Narrative feedback was left by almost all responding patients, to least one of the open-ended questions. The majority of their feedback was positive. Several patients wrote their stories about healthcare professionals and workers, also referring to them by name. If patients could not remember names, they sometimes referred to the other details, such as the healthcare workers’ duty shift. Patients often reported specific episodes that can allow learning-by-excellence processes of quality improvement. Such comments highlighted that the compassionate behaviours of healthcare workers made the difference in the hospitalization experience. Feedback can add relevant information regarding the service organization and on staff behaviours, for example:

“*A care worker understood my discomfort without me saying anything… and spontaneously washed my hair.*”

“*As a family member (daughter), I have been allowed to keep our daily habits, like sitting on a chair to have breakfast with my father.”*

“*While I was walking with my husband in the hall, a nurse made my bed. I really appreciated this act of attentiveness.*”

“*The personnel managed my anxiety very well. In particular, a nurse kindly touched my shoulder, giving me attention and a sense of protection. I really thank her..*.”

As shown above, patient comments were related to the positive behaviour not only of the doctors and nurses, but also of the care assistants as well. During workshops and events held for the presentation of preliminary data (Table 1), practitioners highlighted that this kind of results allow them to monitor and value not only their contributions to patient experience, but also those of care assistants. The anecdotal feedback provided specific information on staff and their behaviours that the standard closed-ended questions could not provide.

When sharing these early results with managers and professionals of participating hospitals, both during meetings with managers and during extended workshops, participation and response rates were partially explained by the role of healthcare workers: their being convinced of the usefulness and innovativeness of the initiative, the clarity and quality of communication with patients, and the time they devoted to speaking to patients about the initiative. The importance given to PREMs by the management was reported as being key in creating a positive perception of this innovative survey, and fostering greater trust in its results, which led to healthcare workers being proactive in encouraging patients to share their experience through PREMs. Managers and professionals believe that participation and response rates are higher when healthcare workers take the initiative to promote the PREMs.

Additionally, the creation of a patient contacts list was already a routine activity for the healthcare workers, who already collect such data for other purposes. Therefore, the administrative tasks were not perceived as increasing with PREMs, as stated by the the same healthcare workers who participated in the workshops. This made it possible to implement PREMs as a census-like survey. During workshops and collective events, healthcare professionals also reported improvements over time in the healthcare workers’ ability to inform patients about PREMs. This led also to the creation of a new routine activity, which coul increase participation and response rates over time.

Clinicians and managers recognized the joint and real-time reporting of PREMs data as meaningful, because they have timely access to quantitative measures and qualitative feedback of their own patients at ward and hospital levels. A timely access to the real-time platform was considered crucial: a delayed sharing of PREMs data, due to a delayed distribution of credentials for accessing the platform, was reported as a factor negatively affecting practitioners’ contribution to the PREMs initiative. Not having a timely access to the web platform discouraged practitioners in informing and enrolling patients. Moreover, practitioners indicated that having PREMs data available enhance the staff’s ability to improve patients’ experiences, with respect to waiting times at the admission, communication, team coordination, and other key dimensions of the patient experience, which are measured by the closed-ended standard questions. Additionally, practioners stated that, through an in-depth analysis of the experiences described in the narrative sections, positive and negative issues in daily practices could be confirmed, identified, and addressed. Healthcare managers, particularly at ward level, were sometimes so positively surprised by the quantity of positive feedback from patients that they would immediately report the results to their team. This suggests an immediate gratifying effect of the patients’ positive narration, particularly about competences, skills, kindness and compassion of all the healthcare workers, including the care assistants. In this respect, healthcare professionals and managers recognised the unique possibility, given by this tool, to monitor the behaviours of hospital staff at the lower levels of the organizational structure. Measuring care assistants’ contribution to the positive experience of patients has traditionally been very difficult to monitor. Hence, this innovative PREMs model was recognized by the practitioners as an effective support to people management within hospitals and wards, across all organizational levels. In particular, the innovations implemented in the PREMs allow the identification and acknowledgement of the healthcare workers who were mentioned by patients for the positive difference that their compassionate behaviour made in the patient experience. This makes it possible to convey the importance and value that their work and behaviours had (and can continue to have) to patients, to all the personnel, including care assistants.

### Evolution of the web platform for real-time data reporting

The access to the beta version of the platform was granted, generally, during the first presentations of the preliminary results. This allowed practitioners to contribute in revising the dashboards. Thanks to the healthcare professionals’ involvement, the graphic representation of data evolved in the first months of the initiative. Practitioners can directly and autonomously choose the graphic representation of data among bar, pie and area charts. Stacked bar charts were added into a section of the platform, comparing the patients’ answers to the PREMs questions among suppliers, according to granted permissions (i.e., managers of a LHAs can see data compared among the direct managed hospitals of the LHAs). In addition to the available time-period selection function, a trend visualisation of specific dimensions of patient experience is currently under development, so that changes over time in the patient perception of care quality would be readily visible and quantifiable.

Currently, the narrative comments can be consulted using a key-word research, by open-ended question, or transversally among all the five questions. Some basic statistics were added (i.e., ranking of words’ occurrence) have been added to this section. The development of an algorithm for a real-time sentiment analysis is ongoing, with the goal to integrate it directly into the platform. Other basic statistics were integrated in order to better monitor the initiative (i.e., participation and response rates by ward, trend of these statistics).

## Discussion

According to literature [73], this PREMs survey is feasible and time-effective, by collecting large-scale data and instantly reporting the answers using web-based administration methods. These two aspects are able to overcome key barriers related to the timeliness of data reporting, and to the sampling, which affects the level of data aggregation in their reporting. Indeed, the novelty of the PREMs presented in this study consists of the combination of their well-documented strengths along with innovations aimed at overcoming their limitations and improving PREMs data use [16, 26].

The ongoing initiative, currently implemented in Tuscany and Veneto, continuously collects patient experience data in a systematic way, providing: (i) standard experience data for trend monitoring and benchmarking within and among healthcare organizations and regions, in the multi-dimensional IRPES; (ii) real-time narrative feedback, to bring out episodes, people and behaviours that made the difference to the patients’ experience and that can be used by the healthcare organizations to value people and learn by what worked.

This innovative PREMs model represents the first example of patient survey that achieves such detail, by collecting and reporting patient measures in real-time and up to the ward level. A scaled-down survey instrument [74], like the PREMs here presented, can be used for improving services and for inter-organizational comparisons that are generally only possible in the case of standard national patient surveys [26]. This PREMs model can quickly and effectively ‘close the loop’, by reporting patient feedback up to the ward where they received care [39]. The accessible and user friendly interface of the web platform, and the concise representation of PREMs big data, helps in rendering PREMs results into actionable information [39, 75]. The real-time reporting of data can also allow monitoring and assessment of changes and improvement actions adopted in practice. PREMs representation is a key aspect: the format of data presentation can affect interpretation, usability and actual use of data by practitioners [76]. Practitioners’ involvement has been important for an initial revision of the web platform of data reporting. However, additional research and, particularly, experiments should be done to improve the presentation and communication of data to healthcare professionals, for informing their quality improvement actions. The public disclosure of these data to citizens requires further studied, which should directly involve patients.

The integration in the standard questionnaires of narrative sections allows reporting both qualitative (small stories) and quantitative feedback (big data) [77]. According to literature [39, 41, 78], clinicians found qualitative comments more interesting and relevant than numerical data, in this study. In the narrative feedback, patient take into consideration their whole experience, by reporting on what and who really matters to them; anecdotal information can explain these latter aspects better than close-ended questions [79], especially when patients describe healthcare professionals’ behaviours that made the difference to them. Narrative feedback can bring to light on fundamental aspects of the patient experience, such as compassion and humanity, which are not specifically addressed by closed-ended questions [63, 80]. Compassionate behaviours are relevant behaviours because they impact on the patient compliance to therapies, self-management and outcomes [81–83]. Further research should demonstrate if PREMs positively influence fostering and spreading of compassionate and positive behaviours within hospitals.

In this regard, another potential use of PREMs data concerns the patients’ acknowledgement and value of healthcare workers directly reported by the patients. This was a critical aspect to be addressed in some healthcare organizations of Tuscany and Veneto, where the healthcare workers expressed the need for being more and better valued. According to the practitioners’ feedback, the volume of positive feedback provided by patients can be a lever to encourage, motivate and value clinicians, nurses and care workers. It allows for the monitoring of the care assistants’ contribution to the positive experience of patients, which is usually difficult to measure. Further research could focus on the ability of PREMS to measure and have an impact on healthcare and care assistants’ practice.

With the innovations introduced, the PREMs have moved from the exclusive focus on ‘what’s wrong’, to the recognition and celebration of what worked well and of those who made a positive difference in the experience of patients, in order to make their care even better [61, 84]. A greater emphasis was placed on the identification, recognition and use of positive processes, practices and behaviours, often rare in healthcare management [62], which helps the translation of positivity into desirable outcomes [60, 85]. In particular, the open-ended questions can help identify the positive deviance [86] and activate learning-by-excellence processes within and among healthcare organizations [62, 87], building on the positive and compassionate behaviours recognized by patients. This positive narrative can be a catalyst for quality improvement ideas that could come directly from healthcare workers at all organizational levels [61]. This can lead to a greater willingness and ability to draw lessons from prior experiences and eventually to a positive behavioural change [88, 89].

The integration of PREMs in a performance evaluation system, publicly disclosed and effectively disseminated among healthcare professionals, was proved to have an impact on their daily practice and behaviours, positively affecting the patient experience [4]. In the practitioners’ opinion, the PREMs initiative presented in this paper can contribute in making these behavioural change mechanisms permanent and sustainable, by influencing the healthcare system and personnel at different levels and spreading compassionate behaviours and positive practices within the system. Future research should verify if such a system does actually influence the use of PREMs data by healthcare professionals in implementing patient-driven improvements.

## Conclusion

This paper presents an action research in which researchers and practitioners of two Italian regions worked in designing, implementing and monitoring a PREMs continuous and systematic observatory. The PREMs Observatory exploits to the greatest extent possible the strengths of traditional patient surveys, and addresses several of the issues and limitations in using patient survey data.

According to the preliminary results and practitioners’ feedback, the PREMs initiative presented in this paper can improve the use of PREMs in the day-by-day practice of healthcare managers, professionals and workers in general, and support patient-driven mechanisms of quality improvement, as well as of cultural and behavioural change, due to several innovations that it encompasses. The combination of the PREMs integration into the multi-dimensional performance evaluation system and the operational and positive people management and quality improvement tool is consistent with healthcare managers’ and professionals’ goals of rapidly deploying sustainable quality improvements, by focusing more on an appreciative and positive approach.

## Supplementary information

**Additional file 1.** Questionnaire.

## Data Availability

The datasets analysed during the current study are available from the corresponding author upon reasonable request.

## Abbreviations

PREMs: Patient Reported Experience Measures
IRPES: Inter-Regional Performance Evaluation System
MeS: Management and Healthcare Laboratory
AIOP: Italian Association of Private Hospitals
EMR: Electronic Medical Record
API: Application Programming Interface
LHA: Local Health Authority
H: Hospital (managed by an LHA)
TH: Teaching Hospital

## References

[1] Carman KL, Dardess P, Maurer M, Sofaer S, Adams K, Bechtel C (2013). Patient and family engagement: a framework for understanding the elements and developing interventions and policies. Health Aff.

[2] Coulter A (2006). Can patients assess the quality of health care?: Patients' surveys should ask about real experiences of medical care. BMJ: Br Med J.

[3] Nuti S, De Rosis S, Bonciani M, Murante AM (2017). Rethinking healthcare performance evaluation systems towards the people-Centredness approach: their pathways, their experience, their evaluation. HealthcPap..

[4] Murante AM, Vainieri M, Rojas D, Nuti S (2014). Does feedback influence patient - professional communication? Empirical evidence from Italy. Health Policy..

[5] Hibbard JH, Stockard J, Tusler M (2003). Does publicizing hospital performance stimulate quality improvement efforts?. Health Aff.

[6] Elliott MN, Lehrman WG, Goldstein EH, Giordano LA, Beckett MK, Cohea CW (2010). Hospital survey shows improvements in patient experience. Health Aff.

[7] Bevan G, Evans A, & Nuti S. Reputations count: why benchmarking performance is improving health care across the world. Health Econ Policy Law. 2019;14(2):141–61. 10.1017/S1744133117000561 29547363

[8] Hibbard JH, Stockard J, Tusler M (2005). Hospital performance reports: impact on quality, market share, and reputation. Health Aff.

[9] Browne K, Roseman D, Shaller D, Edgman-Levitan S (2010). Analysis & commentary measuring patient experience as a strategy for improving primary care. Health Aff.

[10] Coulter A, Locock L, Ziebland S, Calabrese J (2014). Collecting data on patient experience is not enough: they must be used to improve care. Bmj..

[11] Zucca A, Sanson-Fisher R, Waller A, Carey M, Boadle D (2017). The first step in ensuring patient-centred quality of care: ask the patient. European journal of cancer care.

[12] IOM (Institute of Medicine) (2001). Crossing the quality chasm.

[13] Wang DE, Tsugawa Y, Figueroa JF, Jha AK (2016). Association between the Centers for Medicare and Medicaid Services hospital star rating and patient outcomes. JAMA Intern Med.

[14] Trzeciak S, Gaughan JP, Bosire J, Mazzarelli AJ (2016). Association between Medicare summary star ratings for patient experience and clinical outcomes in US hospitals. J Patient Exp.

[15] Goldstein E, Farquhar M, Crofton C, Darby C, Garfinkel S (2005). Measuring Hospital Care from the Patients' Perspective: An Overview of the CAHPS® Hospital Survey Development Process. Health Serv Res.

[16] Gleeson H, Calderon A, Swami V, Deighton J, Wolpert M, Edbrooke-Childs J (2016). Systematic review of approaches to using patient experience data for quality improvement in healthcare settings. BMJ Open.

[17] Bombard Y, Baker GR, Orlando E, Fancott C, Bhatia P, Casalino S (2018). Engaging patients to improve quality of care: a systematic review. Implement Sci.

[18] Weinick RM, Quigley DD, Mayer LA, Sellers CD (2014). Use of CAHPS patient experience surveys to assess the impact of health care innovations. Jt Comm J Qual Patient Saf.

[19] Niccolai F, Nuti S (2012). Intensità di cura e intensità di relazioni.

[20] Crawford MJ, Rutter D, Manley C, Weaver T, Bhui K, Fulop N (2002). Systematic review of involving patients in the planning and development of health care. Bmj..

[21] Murante A, Nuti S (2012). The relationship between patient involvement and hospital accreditation standards. Int J Care Path.

[22] DeCourcy A, West E, Barron D (2012). The National Adult Inpatient Survey conducted in the English National Health Service from 2002 to 2009: how have the data been used and what do we know as a result?. BMC Health Serv Res.

[23] Coulter A, Fitzpatrick R, Cornwell J (2009). Measures of patients' experience in hospital: purpose, methods and uses: King's fund London.

[24] Baldie DJ, Guthrie B, Entwistle V, Kroll T (2017). Exploring the impact and use of patients’ feedback about their care experiences in general practice settings—a realist synthesis. Fam Pract.

[25] Groene O, Sunol R (2015). Patient involvement in quality management: rationale and current status. J Health Org Manag.

[26] Flott KM, Graham C, Darzi A, Mayer E (2017). Can we use patient-reported feedback to drive change? The challenges of using patient-reported feedback and how they might be addressed. BMJ Qual Saf.

[27] Coughlan M, Cronin P, Ryan F (2009). Survey research: process and limitations. Int J Ther Rehabil.

[28] Zuidgeest M, Hendriks M, Koopman L, Spreeuwenberg P, Rademakers J. A comparison of a postal survey and mixed-mode survey using a questionnaire on patients’ experiences with breast care. J Med Internet Res. 2011;13(3):e68. 10.2196/jmir.1241 PMC3222165 21946048

[29] Ebert J. F, Huibers L, Christensen B, & Christensen MB. Or web-based questionnaire invitations as a method for data collection: cross-sectional comparative study of differences in response rate, completeness of data, and financial cost. J Med Internet Res. 2018;20(1):e24. 10.2196/jmir.8353 PMC5801515 29362206

[30] Terluin B, Brouwers EP, Marchand MA, de Vet HC. Assessing the equivalence of Web-based and paper-and-pencil questionnaires using differential item and test functioning (DIF and DTF) analysis: a case of the Four-Dimensional Symptom Questionnaire (4DSQ). Qual Life Res. 2018;27(5):1191–200. 10.1007/s11136-018-1816-5 PMC5891556 29468387

[31] Ellins J (2011). Great expectations? Reflections on the future of patient and public involvement in the NHS. Clinical Medicine.

[32] Wright C, Davey A, Elmore N, Carter M, Mounce L, Wilson E (2017). Patients’ use and views of real-time feedback technology in general practice. Health Expect.

[33] Larsen D, Peters H, Keast J. Using real time patient feedback to introduce safety changes. Nurs Manag. 2011;18(6). 10.7748/nm2011.10.18.6.27.c8718 22017150

[34] Graham C, Käsbauer S, Cooper R, King J, Sizmur S, Jenkinson C (2018). An evaluation of a near real-time survey for improving patients’ experiences of the relational aspects of care: a mixed-methods evaluation.

[35] Greaves F, Ramirez-Cano D, Millett C, Darzi A, Donaldson L (2013). Harnessing the cloud of patient experience: using social media to detect poor quality healthcare. BMJ Qual Saf.

[36] Department of Health NHS Choices Team (2013). NHS Choices - Your health, your choice.

[37] Nuti S, Bonini A, Murante AM, Vainieri M (2009). Performance assessment in the maternity pathway in Tuscany region. Health Serv Manag Res.

[38] Otani K (2006). Enrollees' global rating process of health care with the national CAHPS® benchmarking database. Health Care Manag Rev.

[39] Sheard L, Peacock R, Marsh C, Lawton R. What's the problem with patient experience feedback? A macro and micro understanding, based on findings from a three‐site UK qualitative study. Health Expect. 2019;22(1):46-53. 10.1111/hex.12829 PMC6351417 30244499

[40] Riiskjær E, Ammentorp J, Kofoed P-E (2012). The value of open-ended questions in surveys on patient experience: number of comments and perceived usefulness from a hospital perspective. Int J Qual Health Care.

[41] Grob R, Schlesinger M, Parker AM, Shaller D, Barre LR, Martino SC (2016). Breaking narrative ground: innovative methods for rigorously eliciting and assessing patient narratives. Health Serv Res.

[42] Thomé DC, Mid Staffordshire NHS Foundation trust (2009). A review of lessons learnt for commissioners and performance managers following the healthcare commission investigation.

[43] Nuti S, Vola F, Bonini A, Vainieri M (2016). Making governance work in the health care sector: evidence from a 'natural experiment' in Italy. Health Econ Policy Law.

[44] Vainieri M, Lungu DA, Nuti S. Insights on the effectiveness of reward schemes from 10‐year longitudinal case studies in 2 Italian regions. Int J Health Plann Manage. 2018;33(2):e474-e484. 10.1002/hpm.2496 PMC6032864 29380905

[45] Vainieri M, Vola F, Soriano GG, Nuti S (2016). How to set challenging goals and conduct fair evaluation in regional public health systems. Insights from Valencia and Tuscany regions. Health Policy.

[46] Nuti S, Bini B, Ruggieri TG, Piaggesi A, Ricci L. Bridging the gap between theory and practice in integrated care: the case of the diabetic foot pathway in Tuscany. Int J Integr Care. 2016;16(2). 10.5334/ijic.1991 PMC5356204 29042842

[47] Nuti S, Bini B, Ruggieri TG, Piaggesi A, & Ricci L. Bridging the gap between theory and practice in integrated care: the case of the diabetic foot pathway in Tuscany. Int J Integr Care. 2016;16(2). 10.5334/ijic.1991 PMC5356204 29042842

[48] Davies E, Cleary PD (2005). Hearing the patient’s voice? Factors affecting the use of patient survey data in quality improvement. BMJ Qual Saf.

[49] Zakare-Fagbamila RT, Howell E, Choi AY, Cheng TZ, Clement M, Neely M, Gottfried ON. Clinic Satisfaction Tool Improves Communication and Provides Real-Time Feedback. Neurosurg. 2019;84(4):908-18. 10.1093/neuros/nyy137 29669027

[50] Olsen S, Neale G, Schwab K, Psaila B, Patel T, Chapman EJ (2007). Hospital staff should use more than one method to detect adverse events and potential adverse events: incident reporting, pharmacist surveillance and local real-time record review may all have a place. BMJ Qual Saf..

[51] Davies E, Shaller D, Edgman‐Levitan S, Safran DG, Oftedahl G, Sakowski J, Cleary PD. Evaluating the use of a modified CAHPS® survey to support improvements in patient‐centred care: lessons from a quality improvement collaborative. Health Expect. 2008;11(2):160–76. 10.1111/j.1369-7625.2007.00483.x PMC5060434 18494960

[52] Bell J (2014). Doing your research project: a guide for first-time researchers.

[53] Cohen L, Manion L, Morrison K. Research methods in education: Routledge; 2002.

[54] Nuti S, Noto G, Vola F, Vainieri M. Let's play the patients music: a new generation of performance measurement systems in healthcare. Manag Decis. 2018;56(10):2252-72.

[55] Nuti S, Vola F. Amat P. (Eds.). Evaluating the Network healthcare system Performance: 2015 Results of the Italian Regional Collaborative. Pisa: ETS; 2016.

[56] Denscombe M (2014). The good research guide: for small-scale social research projects.

[57] Wallenburg I, Bal R. The gaming healthcare practitioner: how practices of datafication and gamification reconfigure care. Health Informatics J. 2018. 10.1177/1460458218796608. 10.1177/1460458218796608 PMC6769283 30247089

[58] Coulter A, Jenkinson C, Bruster S (2002). The picker patient experience questionnaire: development and validation using data from in-patient surveys in five countries. Int J Qual Health Care.

[59] Rozin P, Royzman EB (2001). Negativity bias, negativity dominance, and contagion. Personal Soc Psychol Rev.

[60] Haizlip J, May N, Schorling J, Williams A, Plews-Ogan M (2012). Perspective: the negativity Bias, medical education, and the culture of academic medicine why culture change is hard. Acad Med.

[61] Shendell-Falik N, Feinson M, Mohr BJ (2007). Enhancing patient safety: improving the patient handoff process through appreciative inquiry. J Nurs Adm.

[62] Kelly N, Blake S, Plunkett A (2016). Learning from excellence in healthcare: a new approach to incident reporting. Arch Dis Child.

[63] Sinclair S, Russell LB, Hack TF, Kondejewski J, Sawatzky R (2017). Measuring compassion in healthcare: a comprehensive and critical review. Patient.

[64] Tocchioni V, Seghieri C, De Santis G, Nuti S (2018). Socio-demographic determinants of women’s satisfaction with prenatal and delivery care services in Italy. Int J Qual Health Care.

[65] Bonciani M, Lupi B, Nuti S. Performance evaluation in healthcare: the experience of maternity pathway from Tuscany to the Italian network of regions. Ital J Pediatr. 2014;40(S1):A35.

[66] Bonciani M, Lupi B (2018). Monitoraggio dell’allattamento materno in Toscana. Seconda parte. Report 2017–2018.

[67] Murante AM, Seghieri C, Brown A, Nuti S (2014). How do hospitalization experience and institutional characteristics influence inpatient satisfaction? A multilevel approach. Int J Health Plann Manag.

[68] Zhang C, Association OH (2008). Hospital e-scorecard report 2008: acute care: patient satisfaction technical summary.

[69] Zaslavsky AM (2001). Statistical issues in reporting quality data: small samples and casemix variation. Int J Qual Health Care.

[70] Marshall MN, Shekelle PG, Davies HT, Smith PC (2003). Public reporting on quality in the United States and the United Kingdom. Health Aff.

[71] Bellè N, Giacomelli G, Vainieri M, Nuti S. Making sense of performance information: an experimental test of the impact of information format on healthcare professionals’ understanding. PAR Under Review.

[72] Italian Data Protection Authority. Regole deontologiche per trattamenti a fini statistici o di ricerca scientifica. Resolution 515 (19 dicembre 2018) https://www.garanteprivacy.it/web/guest/home/docweb/-/docweb-display/docweb/9069637.

[73] Weigl K, Tikk K, Hoffmeister M, De Toni EN, Hampe J, Kolligs F (2019). A web-based survey among adults aged 40–54 years was time effective and yielded stable response patterns. J Clin Epidemiol.

[74] Draper M, Cohen P, Buchan H (2001). Seeking consumer views: what use are results of hospital patient satisfaction surveys?. Int J Qual Health Care.

[75] Woods SS, Evans NC, Frisbee KL (2016). Integrating patient voices into health information for self-care and patient-clinician partnerships: veterans affairs design recommendations for patient-generated data applications. J Am Med Inform Assoc.

[76] Zwijnenberg NC, Hendriks M, Delnoij DM, de Veer AJ, Spreeuwenberg P, Wagner C (2016). Understanding and using quality information for quality improvement: the effect of information presentation. Int J Qual Health Care.

[77] O'Hara JK, Lawton RJ. At a crossroads? Key challenges and future opportunities for patient involvement in patient safety. Qual Saf. 2016;25:565–68. 10.1136/bmjqs-2016-005476 27334867

[78] Wilkinson J. Evidence: Are clinicians engaged in quality improvement?. London: The Health Found; 2011.

[79] Schlesinger M, Grob R, Shaller D, Martino SC, Parker AM, Finucane ML, Cerully JL, Rybowski L. Taking Patients' Narratives about Clinicians from Anecdote to Science. N Engl J Med. 2015;373(7):675. 10.1056/NEJMsb1502361 26267629

[80] Bramley L, Matiti M (2014). How does it really feel to be in my shoes? Patients' experiences of compassion within nursing care and their perceptions of developing compassionate nurses. J Clin Nurs.

[81] Sinclair S, Norris JM, McConnell SJ, Chochinov HM, Hack TF, Hagen NA (2016). Compassion: a scoping review of the healthcare literature. BMC Palliat Care.

[82] Jeffrey D (2016). Empathy, sympathy and compassion in healthcare: is there a problem? Is there a difference? Does it matter?. J R Soc Med.

[83] Sturgeon D (2010). ‘Have a nice day’: consumerism, compassion and health care. Br J Nurs.

[84] Berwick DM. Continuous Improvement as an Ideal in Health Care. N Engl J Med. 1989;320(1):53-6. 10.1056/nejm198901053200110. 10.1056/NEJM198901053200110 2909878

[85] Losada M, Heaphy E (2004). The role of positivity and connectivity in the performance of business teams: a nonlinear dynamics model. Am Behav Sci.

[86] Lawton R, Taylor N, Clay-Williams R, Braithwaite J. Positive deviance: a different approach to achieving patient safety. BMJ Qual Saf. 2014. 10.1136/bmjqs-2014-003115. 10.1136/bmjqs-2014-003115 PMC4215344 25049424

[87] Bradley EH, Curry LA, Ramanadhan S, Rowe L, Nembhard IM, Krumholz HM (2009). Research in action: using positive deviance to improve quality of health care. Implement Sci.

[88] Anseel F, Lievens F, Schollaert E (2009). Reflection as a strategy to enhance task performance after feedback. Organ Behav Hum Decis Process.

[89] Ellis S, Carette B, Anseel F, Lievens F (2014). Systematic reflection: implications for learning from failures and successes. Curr Dir Psychol Sci.

