# Supplementary material for: Using patient-reported measures to drive change in healthcare: the experience of the digital, continuous and systematic PREMs observatory in Italy
Source: BMC Health Serv Res. 2020 Apr 16;20:315. doi: 10.1186/s12913-020-05099-4 (PMC7161006; doi:10.1186/s12913-020-05099-4)
Supplement: Supplementary file 1 — Additional file 1. Questionnaire. [file 12913_2020_5099_MOESM1_ESM.zip › prems _ appendix_ENGLISH_def.docx]

**Appendix – Questionnaire**

The questionnaire have been piloted and used in Italian only.

It is exclusively web-based: all the filters are automatic.

1. Who is filling in this questionnaire? [single response]
   - The patient
   - A parent/custodial parent/legal guardian
   - Another person on behalf of the patient

ACCESS TO THE HOSPITAL

1. What is the main reason why you chose this hospital? [single response]
   - I consider it the best hospital for my health problem
   - My general practitioner suggested it to me
   - The doctor I have chosen for treating my health problem is working there
   - A specialist suggested it to me
   - My relatives or friends suggested it to me
   - It is the closest one to where I live
   - I did not choose it, because I had an urgent attendance at the Emergency Department
   - Other (specify)
2. Is your general practitioner informed about your hospitalization? [single response]
   - Yes
   - No
   - I don’t know
3. [If 3 equal to yes] Did your general practitioner visit you during your hospital stay? [single response]
   - Yes
   - No, but the general practitioner spoke to me on the phone
   - No, he/she did not visit and did not contact me
4. [If 2 not equal to “I did not choose it, because I had an urgent attendance at the Emergency Department”] At the time of admission to the ward, were you welcomed with kindness and courtesy by the ward staff? [Likert scale]
   - Very much
   - Much
   - Enough
   - A little bit
   - Not at all
   - I do not know
5. Do you have any positive or negative comments on the admission to the ward? If so, use this space. [open-ended response]

HOSPITALIZATION EXPERIENCE

1. During this hospital stay, do you think that the ward staff supported you in facing your fears and anxieties? [Likert scale; the question n.7 includes an additional option only for the physiotherapists’ choice: “I was not assisted by physiotherapists”, which filters other questions (marked with a star in this form).]

|  | I did not have any fears and anxieties | Always | Often | Sometimes | Rarely | Never | I was not assisted by physiotherapists |
| --- | --- | --- | --- | --- | --- | --- | --- |
| doctors |  |  |  |  |  |  |  |
| nurses |  |  |  |  |  |  |  |
| physiotherapists |  |  |  |  |  |  |  |

1. During this hospital stay, do you think that the ward staff did everything possible to help you manage your pain? [Likert scale]*

|  | I did not have any pain | Always | Often | Sometimes | Rarely | Never |
| --- | --- | --- | --- | --- | --- | --- |
| doctors |  |  |  |  |  |  |
| nurses |  |  |  |  |  |  |
| physiotherapists |  |  |  |  |  |  |

1. During this hospital stay, did professionals, nurses and the other healthcare workers talk in front of you as if you weren’t there? [Likert scale] *

|  | Never | Rarely | Sometimes | Often | Always |
| --- | --- | --- | --- | --- | --- |
| doctors |  |  |  |  |  |
| nurses |  |  |  |  |  |
| physiotherapists |  |  |  |  |  |

1. Do you have any positive or negative comments on the care you received by the ward staff? If so, use this space. [open-ended response]
2. During this hospital stay, were you involved by the health professionals as much as you would like in the choices related to your care ? [Likert scale]
   - Always
   - Often
   - Sometimes
   - Rarely
   - Never
3. During this hospital stay, were the answers given by the staff to your questions clear? [Likert scale] *

|  | I did not ask any questions | Always | Often | Sometimes | Rarely | Never |
| --- | --- | --- | --- | --- | --- | --- |
| doctors |  |  |  |  |  |  |
| nurses |  |  |  |  |  |  |
| physiotherapists |  |  |  |  |  |  |

1. During this hospital stay, was easy for your family (or someone else close to you) to be informed about your health conditions? [Likert scale]
   - Always
   - Often
   - Sometimes
   - Rarely
   - Never
   - It was not necessary
   - I was alone
   - I don’t know
2. How would you rate the ability of the ward medical and nursing staff to work together? [Likert scale]
   - Very good
   - Good
   - Sufficient
   - Poor
   - Very poor
   - I do not know
3. Was the ward (room, bathroom, corridors, other common spaces, etc.) quiet? [Likert scale]
   - Very much
   - Much
   - Enough
   - A little bit
   - Not at all
4. Was the ward (room, bathroom, corridors, other common spaces, etc.) clean? [Likert scale]
   - Very much
   - Much
   - Enough
   - A little bit
   - Not at all
5. Do you have any positive or negative comments on the comfort of the ward where you stayed? If so, use this space. [open-ended response]
6. Considering the time you/your family could need to organize your return home, how much time passed between the communication of the discharge and the discharge itself? [single response]
   - More than 24 hours
   - Between 24 and 13 hours
   - Less than 12 hours
7. Do you have any positive or negative comments on your hospital experience as a whole? If so, use this space. [open-ended response]
8. Help us give value to the people who took care of you: would you like to indicate any people who impressed you for the way they treated you? If so, use this space. [open-ended response]

DISCHARGE

1. Before you left the hospital, did you receive clear information on…

|  | Completely clear | Clear enough | Not clear | This kind of information was not necessary for me | I did not receive any information |
| --- | --- | --- | --- | --- | --- |
| What to control once back home (i.e. physical activity, food, smoking,…) |  |  |  |  |  |
| What drugs to take once back home |  |  |  |  |  |

1. Before you left the hospital, did you get information in writing on the drugs to take once back home (i.e. duration of the therapy, frequency of administration, …)? [single response]
   - Yes, I received information in writing
   - No, I didn’t receive information in writing, only verbally
   - No, I received no information neither in writing, nor verbally, but they prescribed me drugs
   - No drugs were prescribed to me.
2. Was the discharge letter clear? [Likert scale]
   - Very much
   - Much
   - Enough
   - A little bit
   - Not at all
   - I did not receive the discharge letter
3. Overall, how would you rate the care that you received in the ward? [Likert scale]
   1. Very good
   2. Good
   3. Sufficient
   4. Poor
   5. Very poor
4. How likely is it you would recommend this ward to relatives/friends/acquaintances having with the same health problem as you? [Likert scale]
   - Yes, completely
   - Maybe
   - Not at all

ONCE AT HOME

1. Once back home, did you need home care provided by a nurse or another healthcare worker? [Multiple response]
   - Yes, by someone from the Local Health Authority (LHS)
   - Yes, I paid for someone myself
   - Yes, I needed home care but I did not received any, neither by the LHS nor payed by me
   - No, I did not need any home care
   - No, I was moved to another hospital
   - Other [open-ended]

ABOUT YOU

1. In general, how do you consider your current health status?
   - Excellent
   - Very good
   - Good
   - Very poor
   - Poor
2. Do you have any chronic health conditions? [i.e. heart disease, stroke, cancer, diabetes, chronic respiratory disease, mental illness, muscular-skeletal disorders, digestive system’s disease, visual or hearing impairment, genetic disease]
   - No
   - Yes
   - I don’t know / remember
3. [if 28 equal to “yes”] Was this hospitalization related to a chronic disease that you have?
   - No
   - Yes
   - I don’t know / remember
4. Sex of the patient [single response]
   - Female
   - Male
5. [if 1 not equal to “the patient”] Sex of the respondent [single response]
   - Female
   - Male
6. Birth year of the patient [open-ended response; only 4 numbers starting with 19]
7. [if 1 not equal to “the patient”] Birth year of the respondent [open-ended response; only 4 numbers starting with 19]
8. Highest grade or level of education of the patient [single response]
   - No formal education/Primary school diploma
   - Middle school diploma
   - High school diploma
   - Academic or professional higher education
   - Master and/or doctoral studies
9. [if 1 not equal to “the patient”] Highest grade or level of education of the respondent [single response]
   - No formal education/Primary school diploma
   - Middle school diploma
   - High school diploma
   - Academic or professional higher education
   - Master and/or doctoral studies
10. Citizenship of the patient [single response; the option can be chosen in the list of all countries]
11. [if 1 not equal to “the patient”] Citizenship of the respondent [single response; the option can be chosen in the list of all the countries]
